# Supplementary material for: Study investigating executive function in schizophrenia patients and their unaffected siblings
Source: PLoS One. 2023 Apr 26;18(4):e0285034. doi: 10.1371/journal.pone.0285034 (PMC10132545; doi:10.1371/journal.pone.0285034)
Supplement: Attachment — Submitted filename: Response for Reviewers.docx [file pone.0285034.s003.docx]

Dear Editors and Reviewers:

Thank you for your letter and for the reviewers ’ comments concerning our manuscript entitled “Study investigating executive function in schizophrenia patients and their unaffected siblings ” (ID: PONE-D-22-29812R2). Those comments are all valuable and very helpful for revising and improving our paper, as well as the important guiding significance to our researches. We have studied comments carefully and have made correction which we hope meet with approval. Revised portion are marked in red in the paper. The main corrections in the paper and the responds to the reviewer’s comments are as flowing:

**Responds to the editor ’s comments:**

Thank you for submitting your manuscript to PLOS ONE. After careful consideration, we feel that it has merit but does not fully meet PLOS ONE’s publication criteria as it currently stands. Therefore, we invite you to submit a revised version of the manuscript that addresses the points raised during the review process.

I call out authors’ attention to all reviewers’ comment. But, important as well, to Rev #2 who raised several concerns about the rigour of your Methods (the lack of rigour), eligibility criteria and stats. Please carefully look for studies on the matter that observed illness duration and medication use - since this is *essential to control”. You will find lots of studies on perceptual and high-level that looked this - check their Methods and criteria, not the theme per se.

Response: Thank you very much for your suggestion. We have revised the manuscript according to the reviewer's comments, We have uploaded the minimum data set as supporting Information files, We have controlled for confounding factors and used more rigorous statistical methods to ensure the accuracy of the results. We have supplemented the duration of the disease and drug use in the Methods section and Table 1, The red part represents the modified content.

**Responds to the reviewer’s comments:**

Reviewer #1:

Thank you for addressing reviewers’ concerns. The manuscript is well-written and much better. But I haven’t found the suggested references from my previous reading - to be on Methods and eligibility criteria. Besides, consider Hedges’ g instead of Cohen’s because of the sample size and deviations from kurtosis and skewness. Please correct.

1. Response to comment:

Thank you for your comments.

Based on these observations and in combination with our research, we have cited relevant literature 10.3389/fpsyt.2013.00182,10.1016/j.jpsychires.2022.03.014,10.3389/fpsyg.2018.00288, 10.1097/00004714-200012000-00019.besides, we use Hedges' g instead of Cohen's value.

Reviewer #2:

We will respond based on your comments last time, as there are no new comments for this modification. We uploaded research data and made modifications to the methodology, citing previous research reports, controlling for confounding factors, and making adjustments to drug use.(p4-7)

10. Y M, K O. Cortical gyrification in schizophrenia: current perspectives. Neuropsychiatric disease and treatment. 2018;14. doi:10.2147/NDT.S145273

20. Ohi K, Nemoto K, Kataoka Y, Sugiyama S, Muto Y, Shioiri T, et al. Alterations in hippocampal subfield volumes among schizophrenia patients, their first-degree relatives and healthy subjects. Prog Neuropsychopharmacol Biol Psychiatry. 2021;110: 110291. doi:10.1016/j.pnpbp.2021.110291

1.Response to comment:( There were many careless mistakes, such as typos, inappropriate spaces, inappropriate periods and casual terms, throughout manuscript)

Response:Thanks for your advice, We are very sorry for our negligence of these question. We have revised this, and you can see it in the revised draft

2.Response to comment:（The authors should focus on executive functions but not other cognitive functions or cognitive neuropsychological assessments throughout manuscript.）

Response: Thank you very much for your valuable comments. We use WCST to measure executive function,Use digital span test (forward/backward) (DST F/B), Stroop Test (color/word/interference)(SC/W/I/ T),and Trail Making A/B (TMTA/B) to measure relevant functions is represented in the manuscript by neurocognitive tests which can be discussed to some extent in comparison with the WCST results.

3.Response to comment:( The authors should focus on TE among executive functions throughout manuscript as TE was only different among three groups.)

Response: Thank you for your positive comments, The results part of TE is worth focusing on g; we also tried to find the difference between two, because we selected a group with a lower average age, so it is also a point of interest whether the results are more generalizable. Here, we have mentioned in the revised draft

4.Response to comment:( 4. In Abstract ‘the WCST results demonstrate that the total correct (TC), total error (TE), perseverative response (PR) and perseverative error (PE) scores in the SZ group were significantly lower than those in the HC group (TC (p=0.011), TE (p<0.001), PR (p=0.007) and PE (p=0.002)), and compared to the unaffected siblings’, These directions were incorrect, e.g., TE in the SZ group was significantly lower than those in the HC group?? Definitely incorrect.)

Response: Thank you very much for your comments, and sorry for our misrepresentation. We have revised the abstract section based on the comments of all reviewers(p1,line 25-35)

5.Response to comment:( In Abstract ‘Neurological abnormalities lead to abnormal functioning in siblings and patients, suggesting that genetics plays a considerable role in such results.’ The current study did not examine or focus other cognitive function except for executive function.)

Response: Thank you very much for your comments, The main concern of our study was the difference in executive function between patients and unaffected patients, on the basis of which we measured MOCA, other tests were assessed around executive function, and because of the patients' limited energy, we only measured the patients' basic cognitive function on the measurement task

6.Response to comment:( In introduction ‘Most research indicates that executive impairment is a core feature of cognitive impairment [4].’, Please clarify why the author consider executive impairment is a core feature among cognitive impairments.)

Response: Thank you very much for your advice. Cognitive deficits in executive performance, working memory and attention are considered to be core features in patients with schizophrenia, because these deficits are present from the first psychotic episode^1^. We have reviewed the relevant literature formulation and found that the formulation is not particularly appropriate, so we have adjusted the formulation.

[1] Breton F , A Planté, Legauffre C , et al. The executive control of attention differentiates patients with schizophrenia, their first-degree relatives and healthy controls[J]. Neuropsychologia, 2011, 49(2):203-208.

7.Response to comment:( Several previous studies investigated differences in executive functions assessed by WCST among patients with schizophrenia, unaffected relatives, and healthy controls. The authors should cite these previous studies and summarize these findings. Furthermore, they mention what is novel in this study.)

Response: Special thanks to you for your good comments. In the background section we provide a review of relevant studies on executive functions. And the novelty of our study lies in the fact that we selected a group with a younger mean average age to explore the differences in executive function between patients and unaffected siblings.

8.Response to comment:( However, in many studies, participants have a greater age at onset.’ Please cite references correctly.)

Response: Thank you for your comments, we have made changes to this.

18. Gold JM, Robinson B, Leonard CJ, Hahn B, Chen S, McMahon RP, et al. Selective Attention, Working Memory, and Executive Function as Potential Independent Sources of Cognitive Dysfunction in Schizophrenia. Schizophrenia bulletin. 2018;44: 1227–1234. doi:10.1093/schbul/sbx155

9.Response to comment:(Studies have suggested that the earlier the age at onset is, the more severe the executive function impairment.’ Please cite references correctly.)

Response: Thank you for your comments, We have adjusted the formulation of the paragraph from‘Studies have suggested that the earlier the age at onset is, the more severe the executive function impairment.’ to ‘Studies have suggested that patients with schizophrenia demonstrated similar age-related declines across most neuropsychologic functions ’ .

Sanchez-Torres AM, Basterra V, Moreno-Izco L, Rosa A, Fananas L, Zarzuela A, et al. Executive functioning in schizophrenia spectrum disorder patients and their unaffected siblings: a ten-year follow-up study. Schizophr Res. 2013;143: 291–6. doi:10.1016/j.schres.2012.11.026

Robert Fucetola, Larry J. Seidman, William S. Kremen, Stephen V. Faraone, Jill M. Goldstein, Ming T. Tsuang. Age and Neuropsychologic Function in Schizophrenia: A Decline in Executive Abilities beyond That Observed in Healthy Volunteers. Biol Psychiatry. 2000;48: 137–46. doi:10.1016/s0006-3223(00)00240-7

10.Response to comment:( ‘Second, consistent with previous research,’ Please cite references correctly.)

Response: Thank you for your comments.We have quoted relevant literature.

11.Response to comment:( ‘106 subjects were screened by random sampling in our study’ How did the authors select these subjects by random sampling? ’)

Response: Thank you for your comments, Inclusion or exclusion of groups by random assignment using the random number method among outpatients.

12.Response to comment:(How many siblings and patients were related?)

Response: Thank you for your comments, in our study, A total of 25 family members have relatives with patients.

13.Response to comment:( Why did the authors define as clinically stable using HAMD and HAMA but not PANSS? In general, PANSS.)

Response: Thank you for your valuable comments, in this study, we are collecting patients in psychiatric outpatient clinic, and most of the patients in the outpatient clinic are in the stable stage of the disease, so we will have a professional physician to assess the psychiatric symptoms using PANSS before the start of the experiment, and also to ensure the accuracy of the assessment results, we must ensure that the emotional state of the patients and other participants is stable, so we have chosen HAMA,HAMD in the study.

14.Response to comment:( ‘The unaffected siblings were the brothers or sisters of the patients. The inclusion)

Response: Thank you for your comments, the unaffected sibling in our study was the patient's brother or sister.

15.Response to comment:( criteria for the siblings were as follows: … (3) a history of the long-term use of drugs or mental disorders among the family members.’ Incorrect. The unaffected siblings have a history of schizophrenia among the family members.)

Response: Thank you for your valuable comments, there is a problem with our presentation. ‘a history of the long-term use of drugs or mental disorders among the family members.’ Modify to ‘a history of the long-term use of drugs or mental disorders.’ Because family members with mental illness are not able to be included in the experiment.

16.Response to comment:( They should apply ANCOVA with age and sex as covariates but not ANOVA.)

Response: Thanks for your reminder, we have corrected the statistical method and updated the relevant data(p10,line 4-9).

17.Response to comment:( They should correct for multiple testing)

Response: Thank you for your comments, we have made adjustments to the statistical methods section to ensure the accuracy of the results.(p10,line 1-15)

18.Response to comment:( In results ‘The neuropsychological assessment and the study variables were assessed using a one-way analysis of variance (ANOVA) and χ2 tests. When a difference was observed among the three groups, a post hoc comparison using LSD or Tamhane was performed (Table 1).’, ‘To explore whether the WCST performance of the patients and their siblings is related to the neuropsychological tests (MOCA, Stroop test, TMT A and B), we used Pearson’s correlation analyses.’ ‘To explore whether the WCST scores of the patients is related to their Clinical symptoms (PANSS total, PANSS positive, PANSS negative), we used Pearson’s correlation analyses.’ These sentences should be replaced to methods section.)

Response: Thank you for your suggestions, we have made adjustments to these sections.

19.Response to comment:( ‘Compared with their siblings, we found significant differences in the patients in some variables as follows: SCT [p=0.037] and SWT [p=0.034]; TMT B [p =0.028]), SCT [p= 0.033], SWT [p=0.013], SIT [p=0.001], DSTF (p < 0.001), DSTB (p=0.004), TMT A [p=0.008] and TMT B [p= 0.003] showed differences between the healthy controls and unaffected siblings.’ Please check the sentence carefully. differences in the patients?)

Response: Thank you for your suggestion, we have corrected this.(p11,line 8-13)

20.Response to comment:( ‘The data displayed in Table 2 show the results of the WCST.’ Fig2?)

Response: We are very sorry for the trouble caused to your review, Fig2 may have a problem when uploading the file, we will pay attention to this problem in the revised manuscript, Fig2 shows the difference between the three groups of subjects.

21.Response to comment:( ‘In any of the three groups, no relationship was found between the WCST performance and the demographic information.(Table 2)’ Table 2?)

Response: Thank you for your suggestion, the demographic data analysis is shown in table3, the sentence was misrepresented and we have removed it.

22.Response to comment:( Overall, I did not check these mistakes throughout manuscript because there were too many mistakes.)

Response: Thank you for your comments on the manuscript during your busy schedule, and we apologize for our mistakes. At the same time, we are doing our best to improve the manuscript.

Thank you very much for your comments. The quality of our manuscript has been improved, and we hope to receive your recognition

Reviewer #3:

Thank you for your precious opinions

Response to comment: (The authors have revised and improved the article. They have studied comments carefully and have madecorrection. They have uploaded the minimum data set as supporting Information files, controlled the confounding factors, updated the results, and added relevant documents, made corrections in the method section, results and discussion.)

Response: Thank you very much for your comments. Due to your suggestions, the quality of our manuscript has been improved. On this basis, we have added more valuable research literature. Thank you very much for your valuable comments.
